# Cardiomyocyte Calcium Ion Oscillations—Lessons From Physics

**DOI:** 10.3389/fphys.2020.00164

**Published:** 2020-02-28

**Authors:** Ohad Cohen, Samuel A. Safran

**Affiliations:** Department of Chemical and Biological Physics, Weizmann Institute of Science, Rehovot, Israel

**Keywords:** cardiomycoyte, calcium, biological physics, oscillations, coarse-grained theory

## Abstract

We review a theoretical, coarse-grained description for cardiomyocytes calcium dynamics that is motivated by experiments on RyR channel dynamics and provides an analogy to other spontaneously oscillating systems. We show how a minimal model, that focuses on calcium channel and pump dynamics and kinetics, results in a single, easily understood equation for spontaneous calcium oscillations (the Van-der-Pol equation). We analyze experiments on isolated RyR channels to quantify how the channel dynamics depends both on the local calcium concentration, as well as its temporal behavior (“adaptation”). Our oscillator model analytically predicts the conditions for spontaneous oscillations, their frequency and amplitude, and how each of those scale with the small number of relevant parameters related to calcium channel and pump activity. The minimal model is easily extended to include the effects of noise and external pacing (electrical or mechanical). We show how our simple oscillator predicts and explains the experimental observations of synchronization, “bursting” and reduction of apparent noise in the beating dynamics of paced cells. Thus, our analogy and theoretical approach provides robust predictions for the beating dynamics, and their biochemical and mechanical modulation.

## 1. Introduction

The heart is an extraordinary organ. From several weeks after conception, and throughout its entire life, the heart constantly beats, generating considerable stresses and strains on its tissues and cells (Hill and Olson, 2012). The individual muscle cells (called cardiomyocytes) that comprise the heart generate contractile forces (Engler et al., 2008; Hersch et al., 2013; Dasbiswas et al., 2015; Nitsan et al., 2016) that translate to relatively large periodic deformations of the heart, i.e., beating. Contraction of adult cardiac cells in tissue is highly regulated by pacemaker cells (Huxley, 1974; Hill and Olson, 2012), which produce electrical impulses that are transmitted to cardiomyocytes, signaling them to contract (Hill and Olson, 2012). The pacemaker cells, unlike adult cardiomyocyte cells, show spontaneous contraction-relaxation cycles even in the absence of an external electrical signal (Vinogradova et al., 2004, 2006; Maltsev and Lakatta, 2007). These cells beat at a relatively fixed frequency, between 0.5 and 3 Hz depending on the species (Kehat et al., 2001; Yang et al., 2002; Majkut et al., 2013). We note that embryonic and neonatal cardiomyocytes also show spontaneous beating, even when cultured as isolated cells (Engler et al., 2006; Tang et al., 2011; Nitsan et al., 2016).

Cardiac contraction is driven by a rise in cytoplasmic calcium ion concentration ([Ca^2+^]) that is coupled to the mechanical contractions of the heart cell (Bers, 2001, 2002). In muscle cells, [Ca^2+^] in the cytoplasm is usually maintained at a relatively low concentration compared with [Ca^2+^] outside the cell and the [Ca^2+^] in a membrane-enclosed organelle called the sarcoplasmic reticulum (SR) (Ibrahim et al., 2011). Oscillations in cytoplasmic [Ca^2+^] are driven by the exchange of calcium ions between the cytoplasm, the extracellular environment and the SR, which is achieved by numerous types of ionic channels and pumps embedded in the extracellular membrane and the SR (Eisner et al., 2000; Bers, 2002; Reed et al., 2014). Activation of a cardiac muscle cell is usually induced by fluctuations or changes in ion channel and pump activity, which cause an influx of calcium ions into the cytoplasm from either the extracellular environment or the SR (Maltsev and Lakatta, 2007). Calcium RyR channels embedded in the SR membrane (Bers, 2002) usually open and close stochastically (with a probability heavily biased toward the closed conformation), but when the calcium in the vicinity of the SR binds to their cytoplasmic side, it forces a conformational change that increases the opening probability (Bers, 2002; Fill and Copello, 2002). Opened RyR channels release calcium stored in the SR into the cytoplasm in a process known as calcium-induced-calcium-release (CICR) (Bers, 2002). After a certain amount of calcium is released, RyR channels revert to their pre-bound dynamics, and ionic pumps restore the cytoplasmic [Ca^2+^] to its baseline value (Huxley, 1974; Bers, 2002).

**Figure 1:**
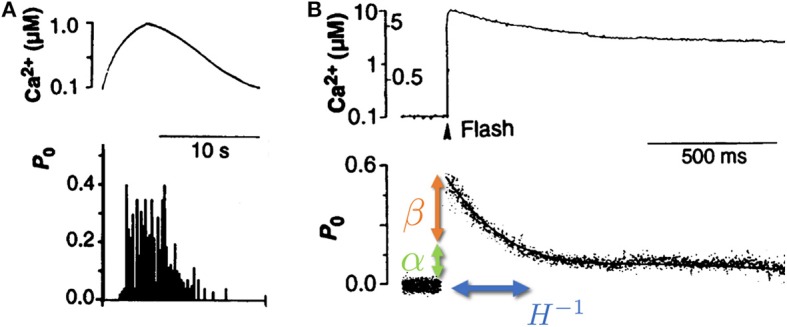
Isolated RyR channel opening probability *P*_0_ as a function of cytoplasmic calcium concentration Ca^2+^. **(A)** When the cytoplasmic calcium concentration (top) was varied slowly (on a scale of ~10 s), the RyR opening probability (bottom) was shown to follow the instantaneous [Ca^2+^]. **(B)** When the calcium concentration (top) was increased rapidly (~1 μs) and kept roughly constant afterwards, the RyR opening probability (bottom) displayed a rapid increase (overshoot, denoted by β—orange arrow), followed by a slow relaxation (with typical rate of ~10 Hz, denoted by *H*—blue arrow) to a new steady-state determined by the long time calcium concentration (denoted by α—green arrow). Adapted from Valdivia et al. (1995). Reprinted with permission from AAAS.

We focus here on the unique role of RyR channels in the cytoplasmic [Ca^2+^] cycle, since recent studies have shown that calcium ion oscillations in pacemaker cells can occur independently of calcium entry across the surface membrane (Vinogradova et al., 2004, 2006; Maltsev and Lakatta, 2007). An important observation is that RyR channels show a time dependent response to changes in Ca^2+^ concentration (see Figure 1) (Valdivia et al., 1995). In these experiments, isolated RyR channels were incorporated in a synthetic membrane, and the Ca^2+^ on the “cytoplasmic” side was dynamically controlled externally with high precision. When [Ca^2+^] was varied slowly (with a time scale of ~10 s), the opening probability of the channel followed the instantaneous [Ca^2+^], in what is referred to in the physics literature as an “adiabatic process” (Risken, 1984) (see Figure 1A). However, when Ca^2+^ was rapidly increased and held constant (a “step function” increase), the opening probability showed an “adaptive” response, an initial sharp increase (overshoot) followed by an exponential relaxation to a steady-state value, with a typical time-scale of ~100 ms (Valdivia et al., 1995) (see Figure 1B). This response suggests that the channel dynamics depends not only on the instantaneous calcium concentration, but also on the rate at which it changes. The adaptive response (along with calcium pump activity) turns out to be a crucial component in the generation of spontaneous calcium oscillations (Cohen and Safran, 2019).

## 2. Minimal Model of Calcium Dynamics

**Figure 2:**
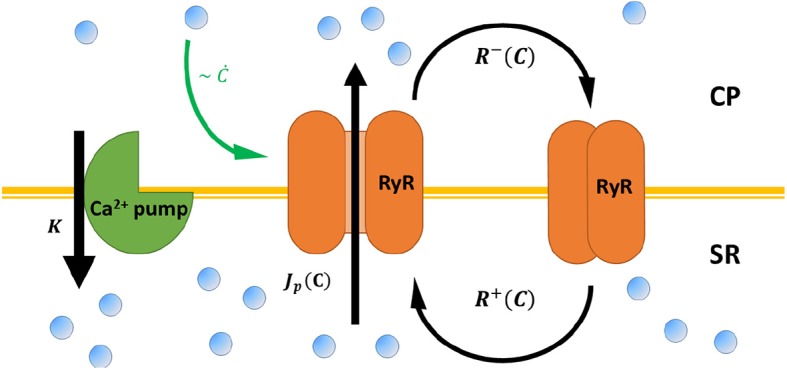
A schematic representation of calcium cycling between the cytoplasm (CP) and the sarcoplasmic reticulum (SR), according to the model presented in Cohen and Safran (2019) and the main text. RyR channels (orange) embedded in the SR membrane stochastically switch between closed (right) and opened (middle) conformation—with rates *R*^±^[*C*] that depend on cytoplasmic calcium *C*. Opened RyR release calcium to the cytoplasm with a current that, in principle, also depends on cytoplasmic calcium *J*_*p*_[*C*]. Calcium is restored to baseline concentrations via calcium pumps embedded in the SR (green, left), the mitochondria, and the cellular membrane (not shown) with a “lumped” rate *K*. RyR “adaptive” response to calcium (see Equation 1) is marked by a green arrow.

Previous models of calcium dynamics (Dupont et al., 1991; Wilders et al., 1991; Atri et al., 1993; Tang and Othmer, 1994; Keizer and Levine, 1996; Jafri et al., 1998; Höfer, 1999; Sneyd et al., 2017) focus on the short time, molecular details of the coupled, multi-component kinetic processes that underlie [Ca^2+^] oscillations. A key feature of many of those models are slow regulatory processes (Atri et al., 1993; Keizer and Levine, 1996; Jafri et al., 1998; Sneyd et al., 2017). As we show below, these effectively cause a time delay in the response of RyR-calcium channels to changes in cytoplasmic [Ca^2+^]. While these models can numerically reproduce many of the features of calcium oscillations, it is difficult to obtain intuition as to *why* myocytes can spontaneously beat in the first place, and how the onset and frequency of beating scale with the characteristic biophysical rates of the system. In contrast, we have recently shown that a minimal model that accounts only for the adaptive RyR dynamics coupled to calcium pump activity (Cohen and Safran, 2019) predicts, in a simple manner, spontaneous calcium oscillations (see Figure 2 for schematic representation). As we review below, these dynamics can be mapped onto a single oscillator equation, with coarse-grained parameters that effectively encapsulate the microscopic dynamics.

The model focuses on the two most relevant degrees of freedom, the opening probability of the RyR channel [denoted by *P*(*t*), with an average value $\bar{P}$], and the cytoplasmic [Ca^2+^] concentration [denoted by *C*(*t*) with an average value $\bar{C}$]. The average [Ca^2+^] concentration reflects a kinetic balance of channels that increase cytoplasmic [Ca^2+^], and active pumps that remove calcium from the cytoplasm (to the SR and or elsewhere). Note that the average [Ca^2+^] concentration can include contributions from an influx of calcium from outside of the cell (via channel activity, or thermal fluctuations), but these are not necessary to generate spontaneous oscillations [as also shown by experimental studies (Vinogradova et al., 2004, 2006; Maltsev and Lakatta, 2007)]. The dynamics of the RyR channels are written in the most general form as:

(1) $$\begin{array}{l} Ṗ=R^{+}\left[C\right]\;\left(1-P\right)-R^{-}\left[C\right]\;P \end{array}$$

where *R*^±^[*C*] are opening and closing rate of the channel that depend on [Ca^2+^] in a general manner. These rates are much faster [~100 Hz (Jafri et al., 1998; Fill and Gillespie, 2018)] than the typical timescale of [Ca^2+^] oscillations (~1 Hz). This allows us to expand Equation (1) around steady-state, and to integrate it over time. The result is an expression for the deviations of *P* from its average $p=\left(P-\bar{P}\right)$, as a function of the deviation of [Ca^2+^] from its average value $c=\left(C-\bar{C}\right)\;$:

(2) $$\begin{array}{l} p\left(t\right)\approx \alpha \bar{C}\;c\left(t\right)+\beta \int_{-\infty }^{t}ċ\left(t^{\prime \prime }\right)e^{-H\left(t-t^{\prime \prime }\right)}dt^{\prime \prime } \end{array}$$

The first term in the right-hand-side of Equation (2) accounts for the response of the RyR channel to changes in [Ca^2+^] concentration relative to steady-state, with a proportionality constant α that reflects the changes of the rates *R*^±^ with [Ca^2+^] around steady-state. The second term accounts for the “adaptive” response of the channel to changes in the [Ca^2+^] concentration, with a magnitude proportional to β that quantifies the overshoot in the channel response to fast changes in the [Ca^2+^] concentration. Equation (2) encapsulates the “adaptive” response observed in experiments (Valdivia et al., 1995). When [Ca^2+^] is slowly varied on the cytoplasmic side of the channel (ċ ≪ 1), the first term dominates, and the opening probability follows the instantaneous [Ca^2+^] concentration (an “adiabatic” change—see Figure 1A). However, if [Ca^2+^] is varied quickly (ċ ≫ 1), the opening probability overshoots by a factor β, and then slowly relaxes to its “adiabatic” value with a typical rate *H* ~ 10 Hz (Valdivia et al., 1995) (see Figure 1B). The “adaptive” RyR response to calcium than enters into the kinetic equation for cytoplasmic [Ca^2+^]:

(3) $$\begin{array}{l} Ċ=J_{p}\left[C\right]\;P\left(t\right)-K\;C\left(t\right) \end{array}$$

The first term on the right-hand-side accounts for the calcium released from the SR into the cytoplasm, where *J*_*p*_[*C*] is the calcium current through the open RyR channels which is in general also a function of cytoplasmic [Ca^2+^] (Bers, 2002). The second term accounts for the various process [SERCA pumps, membrane bound Na^+^–Ca^2+^ pumps and mitochondrial Ca^2+^ uniports activity (Bers, 2002)] that work to return [Ca^2+^] to its steady-state concentration, with *K* the coarse-grained, effective pump-rate at which calcium is restored. Note that since calcium is pumped to the cellular environment, or back into the SR, *K* represents an active process that works against the concentration gradient and as such, requires energy in the form of ATP hydrolysis (Eisner et al., 2000; Bers, 2002). Expanding Equation (3) again, around steady-state, inserting Equation (2) for the RyR dynamics and utilizing again the separation of time scales between the adaptive response (*H* ~ 10 Hz) and the timescale of [Ca^2+^] variation (~1 Hz), allows us to derive a single equation for the [Ca^2+^] dynamics (Cohen and Safran, 2019):

(4) $$\begin{array}{l} m^{*}\ddot{c}+\eta^{*}\;ċ+\Gamma \;c^{2}ċ+\kappa c=0 \end{array}$$

with the coarse-grained parameters derived from the microscopic dynamics:

(5) $$\begin{array}{l} m^{*}=\frac{\beta \bar{J}}{H^{2}},\;\eta^{*}=\left(1-\frac{\beta \bar{J}}{H}\right),\;\kappa =K\left(1-\alpha \right),\\ \Gamma =\gamma m^{*}H \end{array}$$

where $\bar{J}$ is the average current through the channel at steady-state ($\bar{C}$) and the non-linear effect characterized by γ> 0, enters from the expansion of *J*_*p*_[*C*] around steady-state. This represents (Bers, 2002) the tendency of the current to decrease with increasing [Ca^2+^] concentration (for more details see Cohen and Safran, 2019).

Equation (4) is the famous Van-der Pol equation for a non-linear, spontaneous oscillator (Guckenheimer, 1980), with a characteristic frequency $\Omega_{c}\approx \sqrt{\kappa /m^{*}}$. To understand the underlying physics, it is useful to draw an analogy to a classical “spring and mass” system. Consider an object tethered to a wall via a horizontal spring, placed on a frictionless surface, and released from an initial position where the spring is stretched beyond its equilibrium length. The force exerted by the spring causes the object to accelerate toward the equilibrium distance. When it passes that point, the object decelerates till it stops, reverses direction, and continues in the opposite direction, letting the cycle begin again. In short, the object overshoots the equilibrium length of the spring in both the left and right directions.

In a classical system, the first term in Equation (4) would represent inertia—which is the tendency of an object to maintain its velocity. Classical inertia is proportional to the mass of the object, the larger the mass, the larger the overshoot of the equilibrium point. In the chemical system of Equation (4) the “effective mass” m^*^ arises from the overshoot of the steady-state concentration $\bar{C}$ due to the delayed “adaptive” response of RyR channels to [Ca^2+^]. Our model (Cohen and Safran, 2019) based on Equations (1) and (3) predicts that the overshot is proportional to the observed overshoot of the opening probability, β and the channel current $\bar{J}$. The expression for the effective mass in Equation (5), predicts that m^*^ decreases as the rate of adaptation *H* increases. This is because large *H* means that the [Ca^2+^] response quickly returns to its “adiabatic” value so that the overshooting has little effect.

The second term in Equation (4), proportional to η^*^, represents an effective, linear friction. In the classical system friction is proportional to the velocity of the object, and always oppose the motion of the object. Thus, in the presence of friction, the oscillations of the mass decrease in amplitude as time increases and the object asymptotically comes to rest at the equilibrium length of the spring. Similarly, in the chemical system the “effective friction” is proportional to the rate of change of [Ca^2+^]. However, the “effective friction” can switch from regular dissipation (η^*^ > 0) to “negative dissipation” (η^*^ < 0). When the “effective friction” is positive (η^*^ > 0), any oscillations decay over time and the system effectively goes to its steady state concentration at long times. On the other hand, if the “effective friction” is negative (η^*^ < 0), any change in the [Ca^2+^] concentration is amplified by the friction, which would cause a divergence in the [Ca^2+^] concentration as time increases. The origin of the “negative friction” (as one can see from Equation 5) is the same feedback effect that gives rise to the “effective mass.” The increase is eventually saturated by higher order terms in the friction (third term in Equation 4, ~ Γ, see Appendix B of Cohen and Safran, 2019 for one possible derivation), which can be a combination of several microscopic effects (reduction of [Ca^2+^] current with increasing cytoplasmic [Ca^2+^], inactivation of RyR, variation of the adaptation rate etc.). Thus, given the right conditions ($\beta \bar{J}>H$), the activation of RyR by [Ca^2+^] is enough to destabilize the system.

Finally, in the classical system, the fourth term of Equation (4) would represent the restoring force applied by the spring which pulls the object toward the equilibrium distance. This restoring force in our calcium system is proportional to the activity of calcium pumps (*K*), with a correction due to the “adiabatic” response of RyR to [Ca^2+^] (α < 1). It is important to note that, unlike the classical system, the steady-state around which the chemical system oscillates is far from equilibrium (since there is still a large concentration difference between the SR and the cytoplasm). Moreover, the calcium pumps that work to restore cytoplasmic [Ca^2+^] concentrations work against this concentration gradient, and thus require constant input of energy (in the form of ATP). Therefore, in contrast to the classical “passive” oscillator, the chemical oscillator of Equation (4) is inherently active. In the absence of pump activity, thermodynamics predicts the system will equilibrate with the [Ca^2+^] concentration in the SR and the cytoplasm becoming equal.

While further experiments are required to evaluate the parameters of the model, we can estimate those based on previous models for cardiomyocyte oscillations. As discussed above, the adaptation time of an RyR channel was measured as ~100 ms, which translates to *H* ~ 10 Hz (Valdivia et al., 1995). The average current through an RyR channel used in previous models (Dupont et al., 1991; Tang and Othmer, 1994) is on the order of $\bar{J}\sim 20\;\mu M\cdot s^{-1}$. The fact that isolated cells can switch between relaxation and spontaneous oscillations dynamics suggests that those are close to the critical transition of η^*^ = 0. Thus, for oscillating cells sufficiently close to this transition (we take here as an example |η^*^| = 0.1), we estimate β ≈ 0.55 μ*M*^−1^. We thus approximate the effective mass as m^*^ ≈ 0.1 *s*^−2^, which along with a pumping rate *K* ~ 1 *s*^−1^ (Dupont et al., 1991; Tang and Othmer, 1994), and α ≈ 0.2 (estimated from Valdivia et al., 1995) yields an oscillation frequency Ω_*c*_ ~ 2.5 Hz, similar to the frequencies observed in experiments (Tang et al., 2011; Nitsan et al., 2016). Note that even further away from the transition to spontaneous oscillations ($\beta \bar{J}/H\gg 1$), the frequency of oscillations is even lower.

The amplitude of oscillations in our model scales as $\sim \sqrt{\eta^{*}/\Gamma }$ (Cohen and Safran, 2019), which represents the combined effects of both the linear and non-linear friction terms. For the observed amplitudes of ~5 μM around the steady-state (Dupont et al., 1991; Tang and Othmer, 1994), (and for the case of |η^*^| ~ 0.1, close to the transition), one can estimate the non-linear saturation parameter as Γ ~ 0.25 μ*M*^−2^. Note that the microscopic origin of the non-linearity can arise from several different effects (such as saturation of current, direct inactivation of the adaptation, calcium dependence of the adaptation time etc.), and more experiments are required to distinguish between these mechanisms.

## 3. Analytical Predictions of the Model

### 3.1. Response to Periodic Perturbations

Our model which derives Equation (4) from the biophysics of the system, provides an intuitive understanding of the physics of spontaneous [Ca^2+^] oscillations, and how it relates to the kinetics and dynamics of the currents to and from the SR. Moreover, it predicts how the effective parameters (Equation 5) depend on the channel and pump properties and thus, the conditions for the transition from decaying to spontaneous oscillations (η^*^ = 0), and the frequency close to this transition ($\Omega_{c}\approx \sqrt{\kappa /m^{*}}$) (Cohen and Safran, 2019). If we add to Equation (4) an external driving force, we can predicts the response of the system to external perturbations. In the adult heart, [Ca^2+^] oscillations in pacemaker cells effectively determine the heart's beating rate (Hill and Olson, 2012). These oscillations are externally paced by an electrical signal from the brain, which can speed-up or slow down the heart rate in response to oxygen and nutrient demand throughout the body. Pacing spontaneous oscillations is therefore physiologically important in healthy individuals, and any dysfunction may lead to pathological disease (Brodde et al., 1995; Eisner et al., 2000).

It was shown in several experiments that cardiomyocyte beating can be paced electrically (Xia et al., 2000; Radisic et al., 2004; Serena et al., 2009) or mechanically (Tang et al., 2011; Nitsan et al., 2016; Viner et al., 2019). For electrical pacing, the cell is subject to an external electrical field which causes voltage-sensitive ion channels on the cell membrane (or the SR) to open, allowing an influx of ions to the cytoplasm (Berger et al., 1994). For mechanical stimulation, the cell is subject to an oscillating mechanical force (Tang et al., 2011; Nitsan et al., 2016; Viner et al., 2019), which can couple to the cell membrane (or the SR), through integrin adhesions (Peter et al., 2011), or directly affect actomyosin contractility. Effectively, the mechanical deformation translates into a flux of Ca^2+^ ions into the cytoplasm (either from the environment, or from the SR).

**Figure 3:**
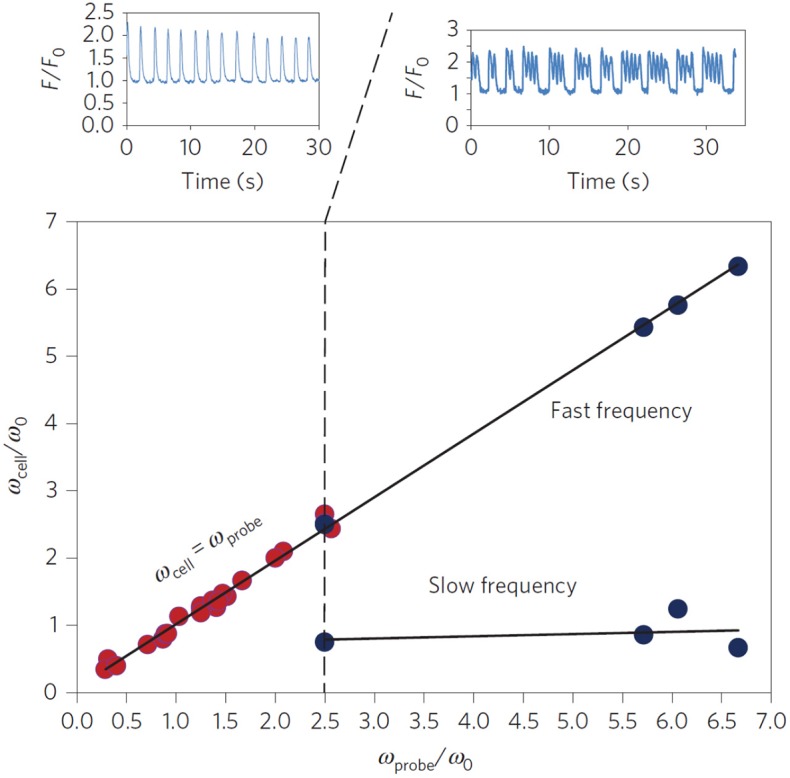
Experimental entrainment of cardiomyocyte beating by a mechanical probe. Isolated cardiomyocytes (*n* = 30) seeded on an elastic substrate were subject to mechanical pacing by an oscillating inert probe located ~100 μm away from the paced cell. The probe introduced periodic deformations of the underlying substrate with a frequency ω_*probe*_. The cell beating frequency was measured before (ω_0_) and ~15 min after (ω_*cell*_) the pacing probe was activated. The scaled cellular beating frequency (ω_*cell*_/ω_0_) was plotted vs. the scaled probe frequency (ω_*probe*_/ω_*o*_) since ω_0_ varies between cells. Red dots represent full entrainment by the probe, i.e., synchronization to the probe frequency (see top left figure, plotting the scaled fluorescent [Ca^2+^] signal *F*/*F*_0_ of a representative cell). Blue dots represent “bursting” behavior, where the cell alternates between beating with the frequency of the probe ω_*probe*_ and quiescence (see top right figure). The time between consecutive intervals of quiescence was comparable to $\omega_{0}^{-1}$. Reprinted by permission from Springer: Springer Nature (Nitsan et al., 2016).

Recent experiments have shown that purely mechanical signals, can control the beating of cardiomyocytes (Tang et al., 2011; Nitsan et al., 2016). In these measurements, nearby neonatal cardiac cells, seeded ~100 μm apart on an elastic gel, synchronize their beating phase and frequency even without direct contact (Tang et al., 2011; Nitsan et al., 2016). By introducing (at similar distances) an inert probe that induced periodic elastic deformations in the substrate, the beating cells were entrained (i.e., synchronized their beating with the deformation of the substrate). All this despite that the cell and probe are not in direct physical contact, or coupled electrically in any way (Nitsan et al., 2016) (see Figure 3). Complete synchronization was observed for a range of frequency differences between the spontaneous and probe frequency. When the difference in frequencies becomes large enough, the cells displays “bursting” behavior, where intermittent periods of synchronized contraction and quiescence are observed. Interestingly, the bursting regime is characterized by several beats at the frequency of the probe, followed by a quiescent interval. The overall duration of the combined beating and quiescence is comparable to the inverse of the spontaneous beating frequency of the cell.

**Figure 4:**
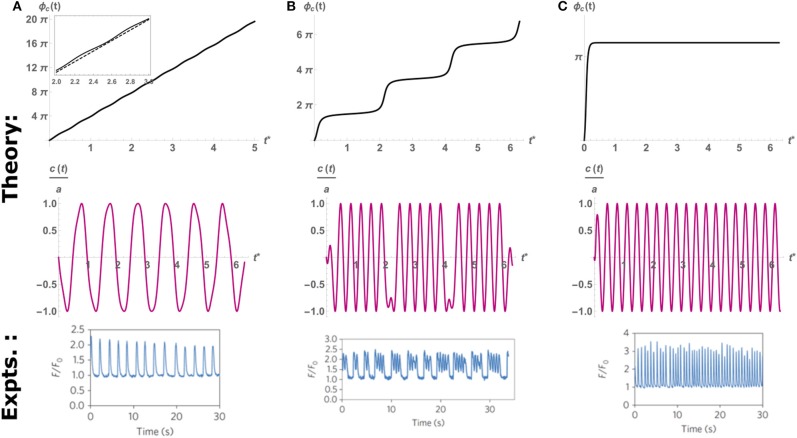
An example of the time evolution of the phase predicted by the theoretical Equation (6) (top row), the resulting scaled oscillation in *c*(*t*) (middle row), and the oscillations observed in experiments (bottom row). Here we fix the cell and probe frequency at Ω_*c*_ = 2π, Ω_*p*_ = 6π, respectively, and vary the amplitude of pacing to control the value of *Q*. **(A)** *Q* = 0.2, below threshold of entrainment (*Q* = 1), the cell beats with its spontaneous frequency Ω_*c*_ as indicated by the quasi-linear increase in phase. **Inset**: comparison to linear slope (dashed) that shows large regions of slips and much smaller intervals of plateaus. **(B)** *Q* = 0.97 Intermittent periods of entrainment (plateaus) followed by fast “phase-slip” events. This corresponds to the “bursting” behavior observed in experiments (bottom row). **(C)** *Q* = 2, above the threshold of entrainment. The cell beats with the probe frequency Ω_*p*_. Top and middle rows adapted from Cohen and Safran (2018). Top and middle rows reprinted by permission from Springer: Springer Nature (Cohen and Safran, 2018). Bottom row reprinted by permission from Springer: Springer Nature (Nitsan et al., 2016).

To account for the effects of external electrical or mechanical perturbations (e.g., the mechanical probe in Nitsan et al., 2016), we supplement Equation (4) on the right-hand-side with external periodic perturbations *f*_*c*_(*t*). We consider here the simple cosine-like perturbation applied in the experiments (Nitsan et al., 2016)—and add to the right-hand-side of Equation (4) a term *f*_*c*_(*t*) = *a*_*p*_ * κ cos(Ω_*p*_*t*), where *a*_*p*_ and Ω_*p*_ are the amplitude and frequency of the perturbations, respectively, and κ is a constant scale factor so that *f*_*c*_(*t*) has the correct dimensions. The periodic external perturbation can entrain (synchronize) the spontaneous beating dynamics of the cell. This can be seen by deriving from the “forced” version of Equation (4), an equation for the dynamics of the beating phase ϕ_*c*_(*t*), defined as difference between the observed beating frequency and the frequency of the perturbation Ω_*p*_ (Cohen and Safran, 2018):

(6) $$\begin{array}{l} {\dot{\phi }}_{c}=\Delta \Omega \left(1-Q\cos\left(\phi_{c}\right)\right),\;Q=\frac{1}{2}\frac{a_{p}\Omega_{c}^{2}}{a_{c}\;\Delta \Omega \;\Omega_{p}} \end{array}$$

where we define the detuning ΔΩ = (Ω_*c*_ − Ω_*p*_), the spontaneous beating amplitude (in the absence of external pacing) $a_{c}=2\sqrt{\eta^{*}/\Gamma }$ (Cohen and Safran, 2018), and the tuning parameter *Q*. Equation (6) is the well-known result by Adler (1946) for the synchronization of coupled electrical oscillators. Spontaneous oscillations occur when the phase changes linearly in time with the detuning ΔΩ (i.e., ${\dot{\phi }}_{c}=\Delta \Omega$), which can only be achieved in the limit of *Q* → 0 (very weak perturbations *a*_*p*_ ≪ *a*_*c*_, or very large detuning ΔΩ ≫ 1, see the top panel of Figure 4A). On the other hand, complete synchronization is achieved when the phase becomes constant at long times (i.e., ${\dot{\phi }}_{c}\to 0$), which means that the cell beats with the frequency of the probe Ω_*p*_. This occurs whenever the tuning parameter *Q* becomes larger than unity (i.e., *Q* > 1), which is the case for large enough probe amplitudes (*a*_*p*_ ≫ *a*_*c*_), or relatively small detuning (ΔΩ ≈ 0) (see Figure 4C). Interestingly, when the tuning parameter is close to its critical value of unity, but is still below the threshold of entrainment (i.e., *Q* ≈ 1), the phase dynamics consist of step-like increases in the beating phase, which translates to “bursting” beating behavior where the cell intermittently switches between beating with the pacing frequency and its spontaneous beating frequencies. This results in a pattern of several beats with the frequency of the probe, followed by short quiescence period, consistent with the experimental observations (see Figure 4B). Thus, the simple dynamics of the paced version of Equation (4) can qualitatively account for the spontaneous, “bursting” and paced dynamics observed in experiments.

### 3.2. Response to Noise

Equations (4) and (6) predict the onset of spontaneous oscillations, and their dynamics when subjected to external pacing in a deterministic manner. However, the beating dynamics of cardiac tissue is not completely deterministic. Irregularities in the beating of the heart have been linked to cardiovascular disease by numerous studies (Kjekshus, 1990; Gage et al., 2001; Benjamin et al., 2018). Arrhythmia (a broad term describing different pathologies associated with increased, decreased, and chaotic heart rate) has been shown to increase the risk of stroke and heart failure (Kjekshus, 1990; Gage et al., 2001). It is therefore crucial that the heart maintain a regular beating pattern to ensure the health of an individual.

While it is reasonable to begin our understanding of the regularity of beating by focusing on an isolated, spontaneously beating cardiomyocyte, we note that single-cell beating dynamics are observed to be more stochastic than those of the entire heart organ (Zaniboni et al., 2000; Nitsan et al., 2016). The stochasticity is manifested in temporal variability of the mechanical stresses exerted by the cell (the amplitude of beating) and of the time between consecutive beats (or the frequency of beating). This variability represent the cumulative contribution of many processes that affect the beating, such as ATP availability, sarcomere structure and alignment, the activity of calcium channels and pumps and many other possible effects (Severs, 2000; Bers, 2002; Kobayashi and Solaro, 2005; Kobirumaki-Shimozawa et al., 2016). Thus, at long times, both the beating amplitude and frequency fluctuate around average values that represents the deterministic, “spontaneous” amplitude and frequency of each cell.

While fluctuations in amplitude affect the maximal stress exerted by an individual cardiomyocyte, these are usually small (~5%) compared to the average amplitude of contraction (Domke et al., 1999; Nitsan et al., 2016). At the organ level, this translates to a roughly constant volume of blood pumped with each beating cycle. Thus, these fluctuations are less important from a physiological point of view. However, fluctuations in the beating frequency (or the time interval between consecutive beats) can range from a few, to tens of percents over time (Domke et al., 1999; Zaniboni et al., 2000; Nitsan et al., 2016). Unlike the fluctuations in amplitude, these deviation can accumulate over time, which translates to the heart cell “skipping” or “adding” beats. These deviations may therefore help understand organ level irregularities, such as arrhythmia.

Recent experiments on isolated, spontaneously beating cardiomyocytes quantify the noise in beating, and its response to external mechanical pacing (Viner et al., 2019). It was shown that cells mechanically paced with amplitudes *a*_*p*_ much lower than those that cause the onset of entrainment (*Q* = 1, in Equation 6) displayed an exponential reduction in the variance of their beating frequency distribution with the pacing amplitude (Viner et al., 2019). This result demonstrates that even when the pacing force is not strong enough to entrain the cell, the introduction of even a weak, oscillating perturbation is enough to increase coherence in the beating of cells (the cells still beat with the spontaneous frequency Ω_*c*_, but with considerably smaller fluctuations).

We have recently shown theoretically and analytically that the reduction in apparent noise can be predicted from the simple model of Equation (4), supplemented by the sum of both an external pacing force [*f*_*c*_(*t*) defined above] and a stochastic noise η(*t*). The noise term is taken to be Gaussian, with average 〈η(*t*)〉 = 0 and temporal correlations 〈η(*t*)η(*t*) = 2*Dδ*(*t* − *t*′) (Cohen and Safran, 2018). The presence of stochastic noise means that there is no deterministic solution and that one must consider the probability distribution of the time dependent [Ca^2+^] concentration. This effectively translated (Hanggi and Riseborough, 1983; Cohen and Safran, 2018) into the probability distribution of the beating phase $P\left[\phi_{c}\right]$:

(7) $$\begin{array}{l} \dot{P}=-\frac{\partial }{\partial \phi_{c}}\left(\alpha^{*}\cos\left(\phi_{c}-\beta \right)P\right)+D^{*}\frac{\partial^{2}P}{\partial \phi_{c}^{2}} \end{array}$$

with $\alpha^{*}\sim a_{p}/a_{c}$ a measure of the relative strength of pacing, β ~ ΔΩ a measure of the phase shift due to the detuning (not to be confused with the adaptation rate β of the previous section), and $D^{*}\sim D/\left(a_{c}^{2}\Omega_{c}^{4}\right)$ a measure of the magnitude of the noise-induced fluctuations of the spontaneously beating amplitude and frequency (i.e., the effective diffusion constant for the phase). Note that in this treatment, we consider the pacing to be below the threshold of entrainment (*Q* ≪ 1). Thus, the beating phase in this case is defined with respect to the spontaneous frequency Ω_*c*_ (and not that of the pacing force Ω_*p*_), and as such is confined to the range 0 ≤ ϕ_*c*_ ≤ 2π.

**Figure 5:**
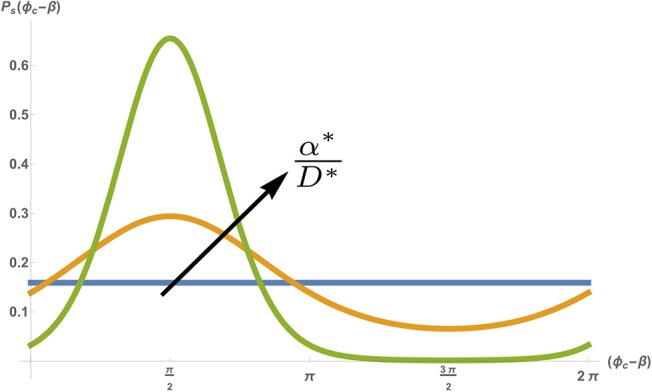
Stationary probability density *P*_*s*_(ϕ_*c*_) as a function of the shifted phase (ϕ_*c*_ − β), for a fixed noise amplitude D^*^ = 0.5 [*s*^−1^], spontaneous frequency $\Omega_{c}=1\;\left[rad\cdot s^{-1}\right]$ and pacing frequency $\Omega_{p}=1.5\;\left[rad\cdot s^{-1}\right]$. Different colors show different pacing amplitudes with α^*^ = 0 (blue) α^*^ = 0.5 [*s*^−1^] (orange) and α^*^ = 2[*s*^−1^] (green). Figure adapted by permission from Springer: Springer Nature (Cohen and Safran, 2018).

Equation (7) describes a diffusion-like process of the phase ϕ_*c*_, but with a drift due to a periodic potential (Risken, 1984). While Equation (7) seems intimidating at first, it can actually be easily understood by examining the ratio α^*^/D^*^, which represents the relative strength of the pacing force compared to the inherent amplitude of the fluctuations. At long times (and given any initial phase ϕ_0_) Equation (7) is expected to go to steady-state ($\dot{P}\to 0,\;P\to P_{s}$). In the absence of any external pacing (α^*^/D^*^ → 0), the inherent noise in the system will cause the phase ϕ_*c*_ to randomly diffuse over time, which means that at very long times the steady-state probability density is flat. This means that in steady-state the phase can take any value between 0 < ϕ_*c*_ < 2π with equal probability. However, when the amplitude of the pacing force is increased (i.e., α^*^/D^*^ increases), the probability density narrows, with an average around 〈ϕ_*c*_〉 = β+π/2 (see Figure 5). Note that a narrowing of the phase does not mean that the cell becomes entrained (i.e., that it beats with the pacing frequency Ω_*p*_). The average beating frequency is still fixed at Ω_*c*_, but the fluctuations around this frequency becomes smaller as α^*^/D^*^ increases. Indeed, in the limit of strong pacing α^*^/D^*^ ≫ 1 (but still below the threshold of entrainment, i.e., *Q* ≪ 1), the probability density approaches an infinitely sharp delta function (~ δ(ϕ_*c*_ − β − π/2)), and we regain the deterministic beating behavior of Equation (4) (with a small effect of the weak pacing force).

## 4. Conclusion

We have demonstrated here the rich set of behaviors observed in the dynamics of spontaneously beating, and paced isolated cardiomyocytes can be captured by a minimal model derived from calcium pumps and channels dynamics. This approach allows for an intuitive understanding of spontaneous beating, and how it reacts to external perturbations. Fundamental understanding of the physics behind cardiomyocyte beating can facilitate the design of better medical treatment in the future. Drawing analogies to well-known physical systems, and understanding how the coarse-grained observables (amplitude, frequency, entrainment, fluctuations in beating) scale with changes introduced to the underlying kinetics, are essential to bridge the gap between the microscopic and the macroscopic view of cardiac beating. The resulting theory is useful in explaining why and how large-scale phenomena (spontaneous beating, entrainment etc.) emerge in a robust manner from the microscopic details. Future analysis should focus upon inter channel coupling within a single cell, and the coupling of calcium oscillations in neighboring cells (using our model as a basis) as a first step to predicting tissue level behavior (Grosberg et al., 2011). Extracting the essence of, and providing simple and testable predictions to the phenomenon of cardiomyocyte beating is an important step in deciphering the riddle of the beating heart.

## Author Contributions

All authors listed have made a substantial, direct and intellectual contribution to the work, and approved it for publication.

### Conflict of Interest

The authors declare that the research was conducted in the absence of any commercial or financial relationships that could be construed as a potential conflict of interest.
